# Digital Health Tools and Patients With Drug Use Disorders: Qualitative Patient Experience Study of the Electronic Case-Finding and Help Assessment Tool (eCHAT)

**DOI:** 10.2196/19256

**Published:** 2020-09-14

**Authors:** Melinda Ada Choy, Elizabeth Sturgiss, Felicity Goodyear-Smith, Gavin JD Smith

**Affiliations:** 1 Academic Unit of General Practice College of Health and Medicine The Australian National University Garran Australia; 2 Research School of Population Health College of Health and Medicine The Australian National University Garran Australia; 3 Department of General Practice Monash University Melbourne Australia; 4 Department of General Practice and Primary Health Care University of Auckland Auckland New Zealand; 5 School of Sociology The Australian National University Canberra Australia

**Keywords:** eCHAT, eHealth, mHealth, digital health, drug use disorders, patient experience, stigma, patient experience, family medicine, general practice

## Abstract

**Background:**

One of the promises of digital health is to better engage patients and improve care for vulnerable populations. Patients with drug use disorders are a vulnerable population who often do not receive the care they need, both for their drug use disorders as well as their other health care needs. Appropriate primary care for patients with drug use disorders needs to be patient-centered, holistic, highly accessible, and engaging. The electronic Case-finding and Help Assessment Tool (eCHAT) was designed as a patient-centered tool for the identification and measurement of problematic health behaviors and mood states.

**Objective:**

The aim of this study was to explore the patient experience of eCHAT at an Australian family medicine clinic for patients with drug use disorders.

**Methods:**

A total of 12 semistructured interviews were conducted with patients, two interviews were conducted with doctors, and one focus group was conducted with patient advocates who were former patients of the clinic where the study took place. The transcripts were analyzed using inductive thematic analysis.

**Results:**

The key themes identified from the interviews and the focus group were as follows: (1) eCHAT helped reduce stigma related to drug use in the doctor-patient consultation, (2) restricted answer options impacted the ability of patients to tell their stories, (3) patient-related response factors, (4) increased efficiency in the consultation process, and (5) divergence in level of concern around security and privacy.

**Conclusions:**

eCHAT has the potential to help vulnerable patients in primary care to engage more with their doctors and reduce experiences of stigma. eCHAT may be a useful digital health intervention in a family medicine clinic for patients with drug use disorders. It has the potential to improve patient engagement and access to health care, which are crucial areas of need in this vulnerable population. However, it is important to clearly communicate the privacy risk of digital health tools and to implement eCHAT such that it will add value to, rather than displace, in-person consultations with the family doctor.

## Introduction

### The Promise of eHealth

The expanding presence of digital health and eHealth is driven by its potential to improve health care outcomes [1]. eHealth refers to the use of internet-based technology for health care and can be used by systems, providers, and/or patients [2]. eHealth is one component of the wider concept of digital health, which is the use of information and communication technology to improve patient well-being and health [3]. Some examples of eHealth used primarily by patients in Australia are electronic medical records, searching for online health information, and booking appointments online.

eHealth has the potential to increase health care access and better engage diverse groups of patients [4]. If eHealth can particularly improve access for vulnerable patients, one key anticipated outcome of increasing eHealth use should be an improvement in health care equity [4].

### The Health Care Needs of Patients With Drug Use Disorders

One example of a vulnerable population affected by health care inequity is people with drug use disorders. They often do not receive adequate treatment support and are more likely to have disability and reduced social and emotional functioning [5,6]. Patients with drug use disorders require customized health care relevant to their drug use situation [5].

Patients with drug use disorders are also more likely to have mental health diagnoses and other chronic diseases [7]. Consequently, patients with drug use disorders often require effective, customized, and sustained general health care, which can be provided through primary care [7].

However, only a small proportion of patients with drug use disorders receive the care they require. One in six people suffering from drug use disorders received treatment for those disorders in 2016 [5]. They also have greater difficulty in access and engagement with general health care services for their family medicine needs [8].

Therefore, the challenge is to design more agile health care services that meet the specific and general needs of patients with drug use disorders [9]. It is crucial that health care services for patients with drug use disorders improve patient engagement and access, as this is a population group with higher health care needs but who also receive proportionally fewer health care services [10].

### Can eHealth Improve Health Care for Patients With Drug Use Disorders?

The critical question is whether eHealth can truly facilitate better engagement and health care access for patients with drug use disorders. The majority of studies involving eHealth and patients with drug use disorders focus on the benefits of telemedicine for treating drug use disorders [11]. Telehealth is a branch of eHealth that creates more *mobile* connections between health care providers and patients [12]. A number of systematic reviews suggest that telemedicine, as well as other eHealth tools, can be effective for improving drug use disorders and creating patient satisfaction [11,13,14].

However, there is scarce literature that explores the role of eHealth in the general health care needs of patients with drug use disorders, particularly in the context of family medicine. Further, there is minimal research into the patient experience of, and engagement with, eHealth in those with drug use disorders. For a patient population with drug use disorders, it is particularly important to understand the patient experience of new eHealth interventions, as they engage health care services less frequently and sometimes with difficulty [8].

One potential example of eHealth in the family medicine setting is the electronic Case-finding and Help Assessment Tool (eCHAT), created in New Zealand [15]. eCHAT is designed as a patient-centered tool for the identification and measurement of problematic health behaviors and mood states. It is a screening survey completed by patients on a tablet computer in the waiting room of a family medicine clinic, with the results provided to the doctor at point of care prior to the consultation [15]. Pilot studies of eCHAT in New Zealand have had positive results in feasibility and acceptability studies [15]. However, prior to this study, eCHAT had not been trialed in Australia, nor had it been trialed at a family medicine clinic with a focus on vulnerable patients with drug use disorders.

### Objective

Our objective was to explore the patient experience of eCHAT when it was trialed in an Australian family medicine clinic for patients with drug use disorders. We were particularly interested in understanding the impact of eCHAT on the patient’s consultation experience and relationship with their family doctor, as these are key factors for health care access for this patient population.

## Methods

### Procedure

From May to November 2019, receptionists offered patients the option to complete eCHAT before their consultation. For each consenting patient, receptionists entered in their unique clinic identifier number into eCHAT on the tablet computer and then gave the patient the tablet. The patient completed eCHAT in the waiting room for 10 minutes and the information went onto a secure web server.

Patients who completed eCHAT answered questions about their recent drug use, mood, and physical activity. They indicated whether they wished to discuss anything further. The doctor reviewed their patient’s answers by searching the patient’s unique clinic identifier number on eCHAT using their desktop computer, just before initiating the patient-doctor consultation.

### Participants

We undertook semistructured interviews with patients and doctors who had used eCHAT. We also ran a focus group with former patients of the clinic who now worked as patient advocates but had not used eCHAT themselves. On days that the research team visited, receptionists invited patients to participate in the study who were (1) over 18 years old, (2) had the time to start eCHAT before their consultation with their doctor and, (3) had the time to participate in a 30-minute interview after their consultation with their doctor.

We used purposive sampling by actively selecting the most productive sample to answer the research question. We recruited participants seeing a diverse range of doctors, a mixture of established and new patients, and a diverse demographic. We continually reflected on our sample and whom we sought to recruit next in coding meetings.

### Data Collection

The Australian National University Human Research Ethics Committee approved this study. Prior to the interview, participants provided written informed consent and completed a short demographic questionnaire. The interview guide was informed by a systematic literature review. After a pilot interview conducted by the first author (MC), the interview guide was further reviewed by an experienced coauthor (GS). The full authorial team collaboratively contributed to the refinement of the interview guide at coding meetings. Interviews and the focus group were audio recorded and transcribed anonymously by a third-party transcription service. The demographic survey results were recorded in Microsoft Excel. The transcription data were managed in both NVivo 11 (QSR International) and Microsoft Word.

### Data Analysis

The interviews and focus group data were coded and processed by inductive thematic analysis. Two researchers (MC, ES) independently reviewed and coded the first five interview transcripts to ensure intercoder validity and reliability. Three researchers (MC, ES, GS) then met to reflect on and discuss the different codes, early themes, sampling frame, and the interview guide.

Following that, one researcher (MC) coded the rest of the interviews and selected key transcripts for the second researcher (ES) to independently code. Both researchers independently coded the doctor interviews and focus group data. Four coding meetings were held with different arrangements of researchers in order to continually reflect on and resolve disagreement over codes and themes. At the final coding meeting, a final list of themes was developed and agreed upon by all four authors.

## Results

### Participant Characteristics

The demographic characteristics of the patients we interviewed are shown in Table 1. The doctors who were interviewed included one male and one female participant. The focus group with patient advocates included three female participants and one male participant.

**Table 1:** Demographic characteristics of patients who were interviewed.

Characteristic		Value (N=12), n (%)
**Gender**		
	Female	2 (17)
	Male	10 (83)
**Highest educational qualification**		
	Lower than year 10	2 (17)
	Year 10	3 (25)
	Year 12	1 (8)
	Trade certification	3 (25)
	Diploma	1 (8)
	Bachelor’s degree	2 (17)
**Occupation**		
	Not employed	4 (33)
	Pension	4 (33)
	Other	4 (33)

### Themes

#### Overview

The key themes from the interviews and focus group were as follows: (1) eCHAT helped reduce stigma related to drug use in the doctor-patient consultation, (2) restricted answer options impacted the ability of patients to tell their stories, (3) patient-related response factors, (4) increased efficiency in the consultation process, and (5) divergence in level of concern around security and privacy.

#### Theme 1: eCHAT Helped Reduce Stigma Related to Drug Use in the Doctor-Patient Consultation

Some patients expressed that it was easier to be comfortable with a screen, compared to a person, when answering questions regarding their drug use and mental health.

Because maybe the person is not comfortable coming up and saying it to their doctor, straight to his face. But if they feel comfortable with that [eCHAT]...

Patient #2, interview

Further, some patients explained that they would be more honest, because interacting with a screen, rather than directly with a person, removed some of the shame associated with their drug use.

I know when I first came to terms with my struggles, I was so ashamed and so guilty I would lie and deny...I still would be so ashamed of why I was there that I would make it seem not as bad as what it actually was. Whereas if I was writing it down—if it was in question form like that—I would just be honest.

Patient #5, interview

One patient explained that the reason that it was easier to be honest with a screen was because a screen is less judgmental than a person.

Whereas with an electronic device, I'm just going to be completely honest with it. I've got no reason to—you know, it’s not going to judge me or expect anything from me.

Patient #3, interview

Patients also felt that their doctors got more information from eCHAT, because eCHAT helped patients themselves tell their stories better. Some patients felt this was because using eCHAT helped them focus on what they wanted to speak to the doctor about.

...it made me separate my thoughts and that just to, it just felt, it made me sort of tell myself something in the doctor's appointment.

Patient #7, interview

Other patients commented that eCHAT gave them more confidence to have a starting point with their story.

...*at least it’s a starting point; you fill out something on there, then it'll give you more reason, it'll make you feel more confident talking to the doctor...* [Patient #6, interview]

#### Theme 2: Restricted Answer Options Impacted the Ability of Patients to Tell Their Stories

A large number of patients commented on how the restricted multiple-choice answer options on the eCHAT questionnaire limited their ability to fully explain their context and situation. Both patients who felt positively and those who felt negatively about eCHAT mentioned that eCHAT’s key weakness was its limited answer options. Particularly in relation to drug use, patients wanted to be able to explain, beyond quantitative questions, about timing frequency.

Everybody will tell you that they smoke too much. Everybody will tell you that. But it’s about “Can you quit, do you want to quit, how come you haven’t?”

Patient #11, interview

Some patients felt that the restricted answers meant that their answers were less likely to be correct.

But I did find that some of the questions, I had to kind of pick one, even though I didn’t feel like there was—I didn’t feel like that was correct, either. It didn’t seem like there was an option that I needed.

Patient #4, interview

One patient commented that the narrow scope of some questions presented an opportunity to be dishonest.

You’ve dabbled in drugs nearly your whole life, but if you’re only answering it the last two weeks, so you just tick that and you go, “Oh, yeah, no worries,” and then, you know, you’re not saying anything.

Patient #9, interview

The idea that eCHAT was not providing a full story frustrated some patients. Some patients felt that this restriction meant that it was far more efficient and satisfying to speak to a doctor directly instead. There was a desire for a qualitative component that would grant the patient more autonomy to co-construct meaning.

It frustrated me that I couldn’t have my own say, I couldn’t, I had to choose one of the options, I couldn’t actually have what actually happened...no one’s going to fit into a little box. Especially someone like me. So I guess when you have little boxes and you don’t have one you fit in, it gets frustrating after a while.

Patient #1, interview

Other patients felt that the restricted answers amplified stigma by placing everyone in the same box based on drug use, rather than understanding the context, story, and factors that mediate the practices of each person.

Well, the government puts everyone like us in the same box. We’re addicts or whatever we are—we've been to jail...I think we should all be judged by our own merits—our own problems and that—and we shouldn’t be boxed together.

Patient #2, interview

#### Theme 3: Patient-Related Response Factors

Some patients had physical disabilities, mostly related to eyesight, that affected their ability to fill out the eCHAT questionnaire. A few patients felt emotionally confronted by some of the questions. One patient started crying when questions were asked about depression, and another patient felt that some of the questions about drug use raised traumatic memories.

They all sort of hit a sensitive spot inside. It brings up emotions to answer some of those questions...

Patient #10, interview

Patients who did not have an established relationship with their doctor sometimes felt that eCHAT improved their relationship with their doctor, but no one felt that eCHAT negatively affected their relationship with their doctor. Patients with an established patient-doctor relationship felt that eCHAT made no difference to that relationship.

The doctors reported that eCHAT was useful for more stable patients who had less urgent agendas, because eCHAT was then able to pick up important, but not pressing, information that had not been previously covered.

...from the kind of chaotic life and you’re just dealing with crises, to the...you’ve gradually got someone engaged and you’re starting to work on those things, that’s the group that I think it’s [eCHAT is] probably most useful with.

Doctor #1, interview

The patient advocates who participated in the focus group strongly agreed that a patient’s stage in the recovery process was the most important factor in their willingness to be honest and to benefit from eCHAT.

If they’re very invested in recovery, then they’ll answer it truthfully either way [electronically or in person].

Patient advocate #1, focus group

#### Theme 4: Increased Efficiency in the Consultation Process

Most patients generally felt positive about the potential for eCHAT to harness waiting room time and provide their doctor with information that would direct or streamline the consultation.

Yeah, I think it’s really good. I think it would save heaps of time...Nobody wants to sit in the doctor’s waiting room all day. I think the faster the appointments are, it’s better for everyone. I think that’s what eCHAT would do, is speed up the whole process.

Patient # 5, interview

However, some patient advocates in the focus group felt that eCHAT would actually result in an overall loss of time, as patients would need to explain the meaning of their restricted answers.

I think the time that you save, not having to ask these questions personally in the room, I think there’s a big opportunity to lose that time that you’ve saved by having to qualify all those questions.

Patient advocate #1, focus group

#### Theme 5: Divergence in Level of Concern Around Security and Privacy

Most of the patients interviewed did not have strong security concerns or register any awareness of how their personal data could be misused.

Why would somebody bother hacking into something like that? Yeah, it doesn’t bother me.

Patient #4, interview

In contrast, the patient advocates in the focus group had stronger security and privacy concerns.

I personally don’t do anything electronic if I can help it...because I don’t trust the systems in place. There are too many holes, too many things can go wrong.

Patient advocate #4, focus group

A few patients who did have concerns in general about security were less worried about eCHAT because they trusted their clinic and knew their name did not go into the system. In general, however, the high level of trust in the clinic and lack of security concerns for some patients meant that they were willing to complete the eCHAT questionnaire if it was made mandatory at the clinic, although they were generally uncomfortable with the concept.

Every time? If I had to?...They’ve been very good to me here, and they’ve offered really good services. So I'd be okay with that.

Patient advocate #3, focus group

## Discussion

### Principal Findings

This paper reports on the findings of a qualitative study exploring the patient experience of an eHealth tool in a family medicine clinic for patients with drug use disorders. Some patients felt that the platform helped them to communicate more honestly, completely, and efficiently with their doctor. A few patients also said that being able to communicate more honestly in the past would have allowed them to get help for their drug use disorders earlier and faster.

Many patients commented on the restricted nature of the multiple-choice answer options. Some felt that these restrictions meant that they were not able to tell their story properly and that the interface compressed their capacity to explain and contextualize factors. A few patients found some of the questions asked in eCHAT confronting, both positively and negatively. Some patients had trouble filling in the eCHAT questionnaire due to a range of physical disabilities.

Very few patients voiced concerns about data security and privacy regarding the information they documented. Those that were aware of possible security issues felt limited concern because no identifying details went into eCHAT.

Patients reported that the use of eCHAT before the patient-doctor consultation sometimes had a positive effect on the patient-doctor relationship, sometimes had no effect, but never generated a negative effect. The optional use of eCHAT before the patient-doctor consultation did not appear to negatively impact patient engagement or access to their family medicine–based health care services.

### Strengths

One strength of this study is the focus on the experience of eHealth for patients with drug use disorders. One key way to improve patient engagement with eHealth is to make the patient experience the guiding principle in eHealth tool design [16]. The way that eHealth increases levels of patient engagement is a common theme in eHealth research [17]. Patient engagement is about creating a more active role for the patient and a more collaborative partnership between the patient and doctor, in order to create a greater protagonist role for the patient in their health care [17,18]. A systematic review exploring how eHealth improves patient engagement found that eHealth interventions generally increase the prominence of the patient’s role in their health care [17]. This matches how eCHAT helped patients communicate their situation more effectively, thereby creating a more active role for the patient and improving the collaboration with the health care provider. As patient engagement and disengagement are highly relevant factors among patients with drug use disorders, the ability of eCHAT to moderately increase patient participation and representation in health care is a promising feature of eCHAT for this vulnerable population.

Another strength of this study is that it uniquely explores the role of eHealth in the general health care needs of patients with drug use disorders, particularly in the context of family medicine. While it is already known that eHealth tools can help with the treatment of drug use disorders and may improve patient satisfaction, this shows that eCHAT could improve holistic patient-centered family medicine care for a vulnerable population of patients with drug use disorders [11,13,14]. This study showed that eCHAT helped some patient-doctor relationships to improve and did not negatively affect any patient-doctor relationships.

Our finding that eHealth tools have the potential to reduce what might be interpreted as *transactional stigma*—that is, shame and stigma arising as a consequence of patients confessing their failings to an authority figure—is significant for a patient population with drug use disorders. Patients with drug use disorders are a highly stigmatized and marginalized group, which can reduce their access to health care and social services [19]. If eCHAT can help reduce stigma and improve patient access, it means that eHealth can have a role in improving access to care for this vulnerable group.

This study also illuminated some other stigma-related concerns of patients with drug use disorders when it comes to engaging with eHealth. A phenomenological study of people with drug use disorders found that when patients felt they were listened to properly by nurses, they responded better to treatment programs and experienced reduced feelings of stigma [20]. The importance of being properly listened to may explain why the restricted answers were problematic for this patient group. Therefore, the concern over restricted answers in combination with some of the more emotionally confronting questions in eCHAT make clear that the eHealth tool was best used when paired with the face-to-face patient-doctor consultation. Pairing eCHAT with a face-to-face consultation means that the patient can have extra emotional support but also be presented with the opportunity to explain their situation in more detail so that their drug-taking practices can be better contextually understood.

### Limitations

While the sample size of this study is small, it uniquely explores the patient experience of an eHealth tool in a population of patients with drug use disorders in the context of their family medicine clinic. Further, the additional data from the focus group and doctor interviews serve as a useful compliment and contrast to the patient perspective.

For example, the prominent privacy and security concerns of the focus group members were in stark contrast to the perspectives of the patients. This contrast begs the question of whether the patients, as a consequence of their requiring and seeking health care, were less able to identify and raise critical concerns about potential privacy and data security issues than their more detached and independent patient advocate counterparts. It is possible that the interview setting (ie, the clinic where the patients receive care) and the interviewer (ie, a family medicine doctor from another practice) affected the response of the patient participants during the interviews. Even if this had some impact on the interview data, the reticence of patients to accentuate concerns pertaining to the use and processing of their personal data highlights the need for administrators of eHealth to clearly communicate the potential surveillance, security, and privacy risks related to the use of digital health tools, especially for those who are most vulnerable. Adequate risk communication is particularly appropriate when seeking informed consent for the use of an eHealth tool from patients who require access to health care.

Another example of how the different data worked together was that the concerns raised by patients about the restricted answer options in eCHAT were partly offset by the doctors, who explained that they principally used eCHAT to initiate conversations with their patients.

Implementation issues meant that not many patients completed the eCHAT questionnaire separate from the study taking place, which left doctors feeling that they were limited in their ability to comment broadly on the effect of eCHAT.

### Future Considerations

The challenges of eCHAT that were identified by participants have been discussed with both the family medicine clinic where the study took place and the creators of eCHAT. Regarding implementation at the family medicine clinic, the risks of upsetting patients and miscommunicating the patient story with restricted answer options make it clear that eCHAT works best as an optional process paired with, and on the same day as, the doctor-patient consultation.

Regarding the design of eCHAT, the options of voice-prompted questions for those with disabilities and open-ended questions to counterbalance concerns around restricted answers have been discussed. There are further developed versions of eCHAT that now have an introductory video that patients can watch before completing the eCHAT questionnaire, which greatly assists with clearly and accessibly communicating security and privacy risks to patients.

A future option for long-term evaluation of eCHAT could build on this study with qualitative analysis of the patient experience of eCHAT in vulnerable populations from multiple clinics.

Our findings suggest that further quantitative, qualitative, and mixed methods research into the experience of vulnerable patients when using other digital health tools may assist these tools in contributing to improving health care equity within communities.

### Conclusions

eCHAT has the potential to help vulnerable patients in primary care to engage more with their doctors and reduce their sense of stigma, but attention must be paid to how consent is obtained from patients and how eCHAT is paired with the consultation.

To move this forward, future research will need to explore how other eHealth tools, and how the concept of eHealth in general, affect the experience of health care for patients with drug use disorders. Understanding the situated experiences of eHealth for specific groups of vulnerable populations can help clarify aspects of design and implementation for eHealth tools that are more likely to improve patient access and equity.

Based on this study, eCHAT could be a useful eHealth intervention in a family medicine clinic for patients with drug use disorders. It has the potential to improve patient engagement and access to health care, which are crucial areas of need. However, care must be taken when implementing eCHAT to make the surveillance, privacy, and data security risks clear to patients, and emphasis needs to be placed on how eCHAT should complement, rather than replace, the face-to-face consultation with the family doctor.

## Abbreviations

eCHAT: electronic Case-finding and Help Assessment Tool
